# Chronotypes and their relationship with depression, anxiety, and fatigue among patients with multiple sclerosis in Vilnius, Lithuania

**DOI:** 10.3389/fneur.2023.1298258

**Published:** 2023-11-27

**Authors:** Ieva Jonušaitė, Eglė Sakalauskaitė-Juodeikienė, Rasa Kizlaitienė, Nataša Giedraitienė, Ieva Sereikė, Justina Liutkienė, Gintaras Kaubrys, Dalius Jatužis

**Affiliations:** Clinic of Neurology and Neurosurgery, Institute of Clinical Medicine, Faculty of Medicine, Vilnius University, Vilnius, Lithuania

**Keywords:** chronotype, circadian rhythm, multiple sclerosis, MEQ, HADS, SFQ

## Abstract

**Background:**

Approximately half of patients with multiple sclerosis (PWMS) experience sleep disorders or changes in the circadian rhythm, that may further promote the pathogenesis of multiple sclerosis. As the prevalence of chronotypes among PWMS remains unclear, we aimed to evaluate the prevalence of chronotypes among Lithuanian PWMS; to assess the relationship of chronotypes with depression, anxiety, and fatigue symptoms; and to compare these results with those of healthy controls.

**Methods:**

We enrolled 101 PWMS and 100 healthy controls. We included 130 (64.7%) and 71 (35.3%) women and men, respectively. The median age of all respondents was 39 [interquartile range (IQR) 20.75] years. Participants were assessed using general questionnaire, Horne-Östberg Morningness-Eveningness Questionnaire (MEQ), Hospital Anxiety and Depression Scale (HADS), and Shortened Fatigue Questionnaire (SFQ). Chronotypes were identified based on the total MEQ score.

**Results:**

The average MEQ scores of the PWMS and control groups were 54 (IQR 15.0) and 53.5 (IQR 13), respectively, which indicated the intermediate chronotype. There was no significant between-group difference in the prevalence of chronotypes (*p* = 0.893). In both groups, individuals with moderate evening and intermediate chronotypes showed higher average HADS depression scores (*p* = 0.022). Further, in both groups, the individuals with the evening chronotype showed the highest average HADS anxiety scores (*p* = 0.001). The PWMS group had a higher average SFQ score than the control group (*p* < 0.001). High SFQ scores were more common among PWMS who had the intermediate (*p* < 0.001) and morning chronotypes (*p* = 0.011). The fatigue level was higher among healthy individuals with the evening chronotype (*p* < 0.001).

**Conclusion:**

The most common chronotype for PWMS and healthy controls was the intermediate chronotype. Further, in both groups, higher HADS depression and anxiety scores were associated with the evening chronotype. Fatigue was more commonly found in healthy controls with the evening, and in PWMS - with intermediate and morning chronotypes.

## 1 Introduction

Circadian rhythm disorders are defined as non-conformities between individual sleep habits and a regular sleep model based on the time of day (1, 2). Circadian rhythm disorders affect working efficiency during the daytime and decrease the general quality of life. The suprachiasmatic nucleus (SCN) is the central pacemaker of the circadian timing system and regulates most circadian rhythms in the mammalian body (3–5). However, circadian rhythm regulation is more complicated and involves circadian genes in every cell, which participate in circadian rhythm regulation from the periphery under the control of the central pacemaker (6–8).

An individual’s chronotype refers to the individual’s tendency to sleep at a certain time during 24 h, which is genetically determined by the internal biological clock (9–12). Chronotypes are affected by circadian polymorphisms and environmental factors such as bright light, physical activity, socialisation, and eating habits (13). Chronotypes can be classified into three main categories: the morning type (when there is a natural desire to sleep early in the evening and wake up early in the morning, with the morning being usually the most productive time of the day), the evening type (an individual usually goes to sleep past midnight and wakes up late in the morning or at noon, with the afternoon being the most productive time of the day), and the intermediate type (the most common type [≈60% of the population], which is described as a combination of the morning and evening chronotypes) (14, 15).

The dominant chronotype of infants and preschool-aged children is the morning type. The evening chronotype is prevalent among teenagers and young adults (≈40%) (13). Notably, the evening chronotype is associated with a negative impact on both health and social life (16–19). The negative effects of the evening chronotype could be attributed to the conflict between the social life regimen and individual chronotypes. Intermediate chronotype is typically observed in adults (16). However, the chronotype returns to the morning type with further ageing (14, 20). The diagnosis of circadian disorders of the sleep-wake cycle is usually based on the patient’s complains of insomnia, moreover, the sleep disturbance should be associated with impairment of social, occupational, or other areas of functioning. Horne–Östberg Morningness–Eveningness Questionnaire (MEQ) may be useful in confirming patient’s circadian preference, in addition, sleep log or actigraphy monitoring for 14 days should be also performed to confirm the clinical diagnosis (2). Therefore, it is important to note that the existence of evening or morning chronotype for the patient is not a disease itself, but a biological condition and basis of a disease to occur in the future. If the patient can adapt one’s lifestyle, work, studies, and other social activities to the chronotype, one can be free of any complaints of sleep quality and quantity, and thus have no circadian rhythm disorder.

Chronotypes can be evaluated subjectively [Munich Chronotype Questionnaire (MCTQ), MEQ] and objectively [dim light melatonin onset (DMLO), phase angle of entrainment, free-running circadian period (tau) from an ultradian forced desynchrony protocol] (10). Objective methods are more accurate and specific; however, they require strict laboratory or sleep clinic environments.

Approximately 50% of patients with multiple sclerosis (PWMS) experience sleep disorders, including insomnia, restless legs syndrome, and narcolepsy (21, 22). PWMS may present the extreme morning chronotype, especially those with severe chronic fatigue (23). Additionally, PWMS experience difficulty falling asleep, complain of restless sleep, and wake up earlier in the morning than desired (21). Changes in the circadian rhythm may further promote the pathogenesis of multiple sclerosis (MS); specifically, decreased melatonin levels in PWMS are related to its insufficient antioxidative and anti-inflammatory effects, which results in the production of endogenous free radicals that may contribute to MS progression (24, 25). Sleep homeostasis in PWMS can be affected by various factors, including demyelinating lesions in sleep-wake centres, chronic fatigue, spasticity, nocturia, depression, anxiety, and the use of medications such as disease-modifying therapies or symptomatic treatment (26–28).

However, the prevalence of chronotypes among PWMS remains unclear (15, 22, 29, 30), including in Lithuania. Accordingly, we aimed to evaluate the sleep habits of Lithuanian PWMS and compare them with those of healthy controls; to determine the prevalence and spectrum of chronotypes; further, we aimed to compare depression, anxiety, and fatigue levels between PWMS and healthy controls with different chronotypes.

## 2 Materials and methods

This cross-sectional study was performed between December 2019 and March 2021. We included 101 PWMS from the Vilnius Multiple Sclerosis Center and 100 healthy individuals in the control group. A total of 210 completed questionnaires were collected; among them, 9 (4,29%) were rejected because of incompleteness and 201 (95,71%) were selected for further analysis. All PWMS were in remission, without relapse and corticosteroid or plasmapheresis treatment within ≥2 months. All patients had relapsing MS. The co-occurrence of other neurological or psychiatric diseases was considered as an exclusion criterion.

We used a general questionnaire created by the authors to collect sociodemographic data (age, sex, education, residence, and marital status) and information about sleep habits (self-reported sleep latency, night sleep duration, naps during the day; see Supplementary S1). Before distribution, this questionnaire was reviewed for simplicity, readability, and content. Further, the questionnaire was evaluated by an MS expert and pilot-tested in 10 patients to eliminate questions that were difficult to comprehend.

Additionally, we used MEQ (31) to evaluate chronotypes, the Hospital Anxiety and Depression Scale (HADS) (32) to evaluate anxiety and depression symptoms, and the Shortened Fatigue Questionnaire (SFQ) (33) to assess the severity of fatigue. Moreover, we asked the patients several questions about their disease, duration of MS, sleep environment, and habits. Neurological disability was assessed by the same neurologist using the Expanded Disability Status Scale (EDSS) (34). The chronotype was determined based on the total Horne-Östberg MEQ score, which ranges from 16 to 86 points. A score above 58 indicated the morning chronotype (59–69 points: moderate morning chronotype; 70–86: definitely morning chronotype), while a score below 42 indicated the evening chronotype (16–30 points: definitely evening chronotype; 31–41: moderate evening chronotype) (14, 31). A score of 42–58 points indicated the intermediate chronotype. HADS scores ≥8 points indicated the presence of anxiety or depression symptoms (32). The level of fatigue was assessed by SFQ, which score varied from 4 (min) to 28 (max) points; an SFQ score ≥18 points indicated significant fatigue (33).

This study was approved by the Bioethics Committee of the Vilnius University Hospital Santaros Klinikos (No. 18-429). All participants provided written informed consent. Statistical analyses were performed using the IBM SPSS 19.0 program. The homogeneity of the groups was tested using Levene’s test to assess the equality of variances. Homogeneous and non-homogeneous variances were described by the average and median, respectively. We used Student’s *t*-test or Pearson’s correlation for parametric variables and the Mann–Whitney U test or Spearman’s correlation for non-parametric or non-homogenous continuous variables. One-way analysis of variance (ANOVA) was used for comparisons among the different chronotype groups. Two-way ANOVA, followed by *post hoc* (Least Significant Difference, LSD) testing, was conducted to analyse the effects of both MS and different chronotypes on parametric variables. Categorical variables were evaluated using the chi-squared test. The difference between variables was considered statistically significant if *p* < 0.05.

At the end of the study, all PWMS received sleep hygiene recommendations.

## 3 Results

### 3.1 Demographic and clinical characteristics

We included 130 (64.7%) and 71 (35.3%) women and men, respectively. The median age of all respondents was 39 [interquartile range (IQR) 20.75] years. Table 1 presents the sociodemographic characteristics of both groups. The average duration of MS was 9 (IQR 9.0) years for both men and women; the median EDSS score was 3 (IQR 2.0) (see Table 2).

**Table 1:** Between-group comparisons of the sociodemographic characteristics.

	Sex		Age, years (IQR)	Education		Employment		Relationship status	
	Male	Female		University degree	Secondary school	Employed	Non-employed	In a relationship	Single
PWMS (*n* = 101)	46 (45.5%)	55 (54.5%)	39 (14)	68 (67.3%)	33 (32.7%)	82 (81.2%)	19 (18.8%)	67 (66.3%)	34 (33.7%)
HC (*n* = 100)	25 (25%)	75 (75%)	34 (25)	79 (79%)	21 (21%)	96 (96%)	3 (3%)	70 (70%)	30 (30%)
Total: *n* = 201	71 (35.3%)	130 (64.7%)	39 (20.75)	147 (73.1%)	54 (26.9%)	178 (88.6%)	22 (11.4%)	137 (68.2%)	64 (31.8%)
*p* value	p* = 0.002		p** = 0.002	p* < 0.001		p* < 0.001		p* = 0.293	

HC, healthy controls; IQR, interquartile range; PWMS, patients with multiple sclerosis. *Chi-square test and **Mann–Whitney U test.

**Table 2:** MS characteristics; depression, anxiety, and fatigue symptoms in the PWMS group.

Data	Mean/median value	Descriptive statistics, IQR/±SD
MS duration, years	9	9
EDSS score	3	2
HADS anxiety score	5.5	7
HADS depression score	5	6
SFQ score	22.43	±0.57

EDSS, Expanded Disability Status Scale; HADS, Hospital Anxiety and Depression Scale; IQR, interquartile range; MS, multiple sclerosis; PWMS, patients with multiple sclerosis; SD, standard deviation; SFQ, Shortened Fatigue Questionnaire.

### 3.2 Sleep habits

There were no significant differences in self-reported sleep latencies between PWMS (57.31 ± 5.45 min) and healthy controls (52.15 ± 5.39 min; *t* (184) = −0.67, *p* = 0.503). However, the median sleep latency in PWMS with clinically significant fatigue (SFQ ≥ 18) was 60 min (IQR 90), while that in healthy controls without fatigue symptoms was 30 min (IQR 40) (*U* = 1,058, *p* = 0.014). Sleep latency was positively correlated with HADS depression (*r*(84) = 0.27, *p* = 0.012) and anxiety (*r*(84) = 0.3, *p* = 0.006) scores in PWMS group.

**Figure 1:**
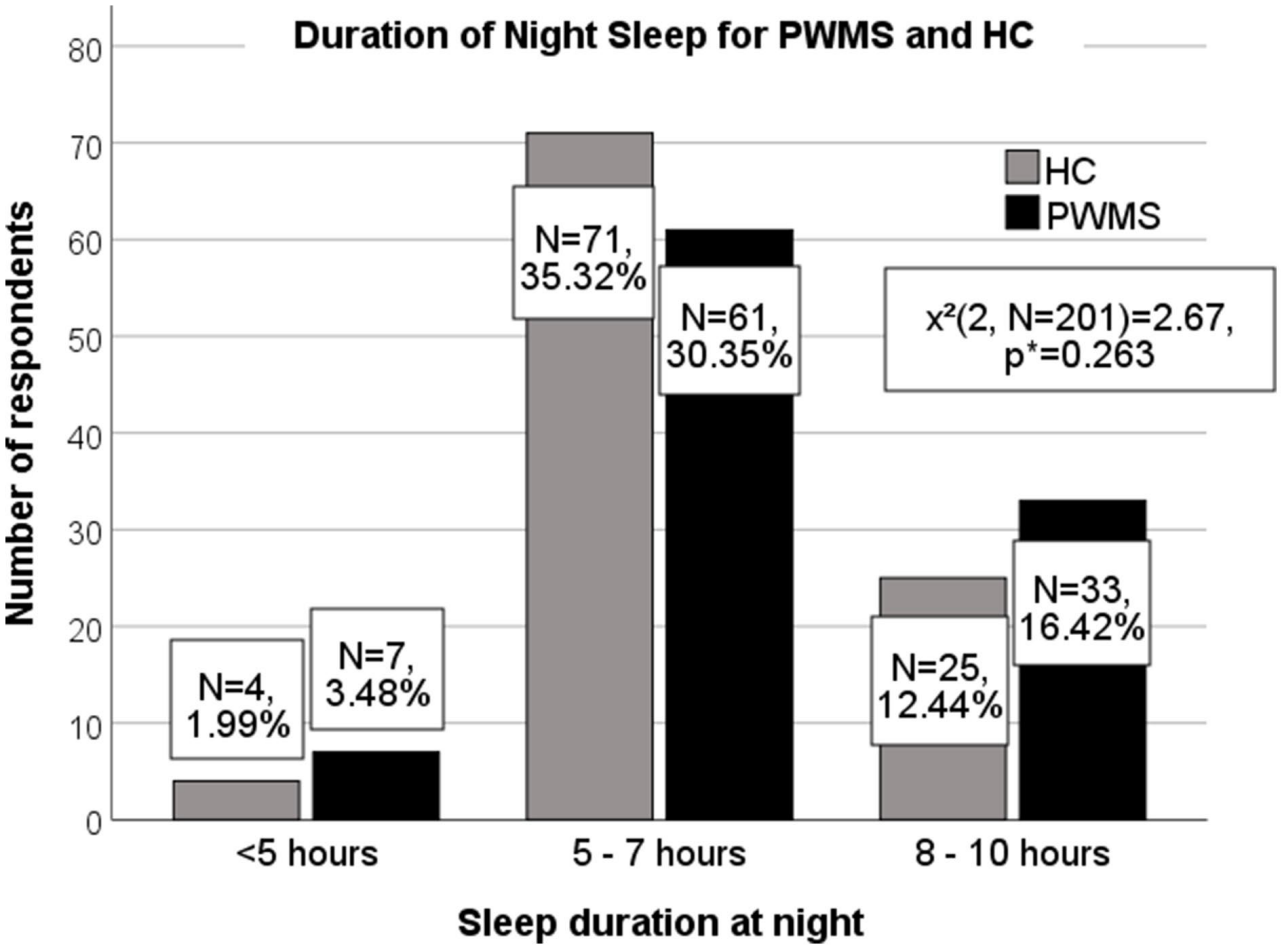
Duration of night sleep among PWMS and healthy controls. PWMS, patients with multiple sclerosis; HC, healthy controls. *Chi-square test.

**Figure 2:**
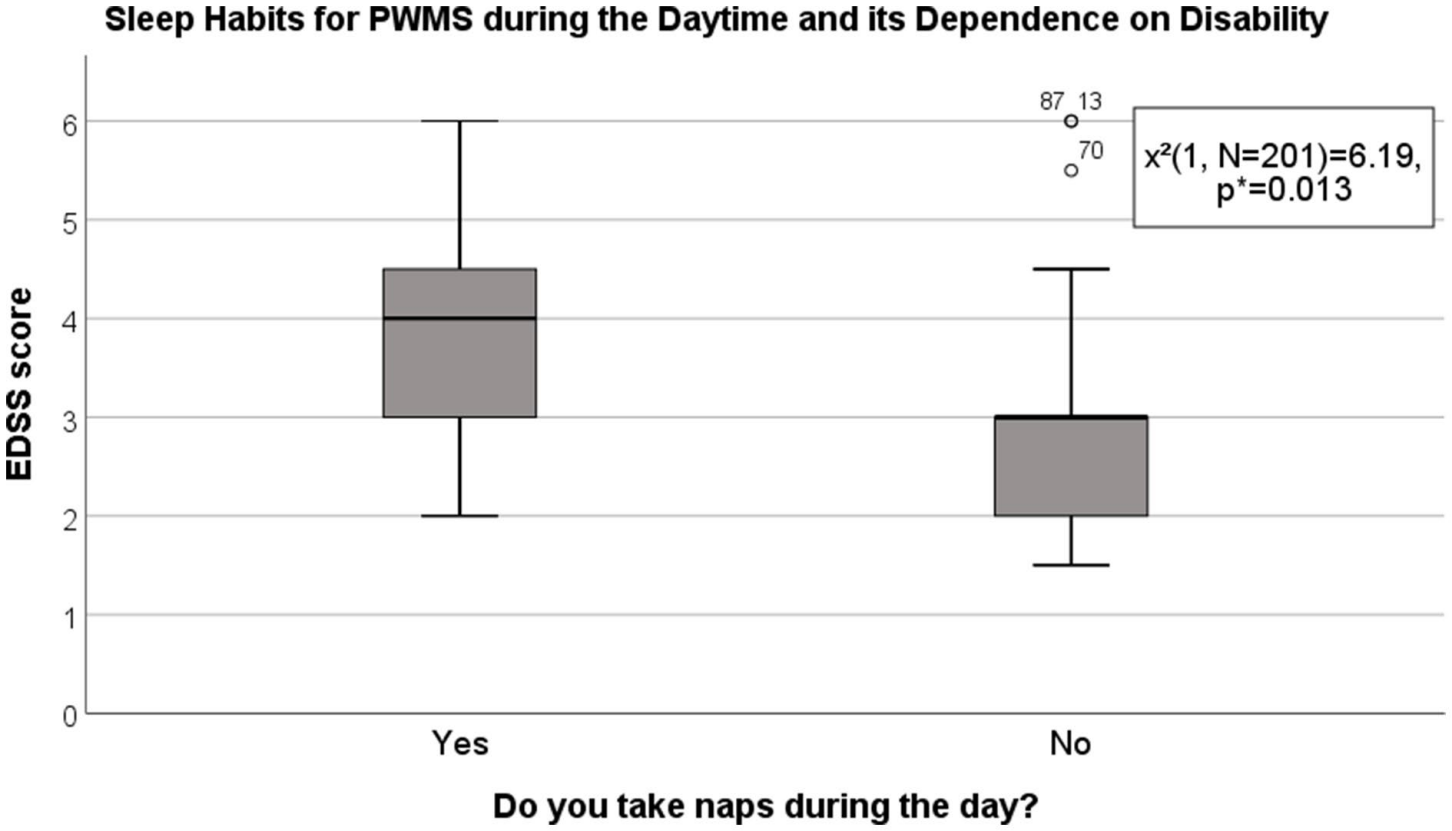
Sleep habits in PWMS during the daytime and their dependence on disability. EDSS, Expanded Disability Status Scale; PWMS, patients with multiple sclerosis. *Chi-square test.

There was no significant difference in self-reported night sleep duration between PWMS and healthy controls (*χ*^2^(2, *N* = 201) = 2.67, *p* = 0.263) (for details, see Figure 1). However, PWMS with clinically significant fatigue (SFQ ≥ 18) slept <5 h per night, compared with healthy controls without fatigue symptoms (14.3% vs 2.4%; *χ*^2^(2, *N* = 103) = 6.68, *p* = 0.036). Among 38 (18.9%) respondents who tended to take naps during the day, 26 (68.42%) were PWMS (*χ*^2^(1, *N* = 201) = 6.19, *p* = 0.013) (see Figure 2).

### 3.3 Chronotypes

**Figure 3:**
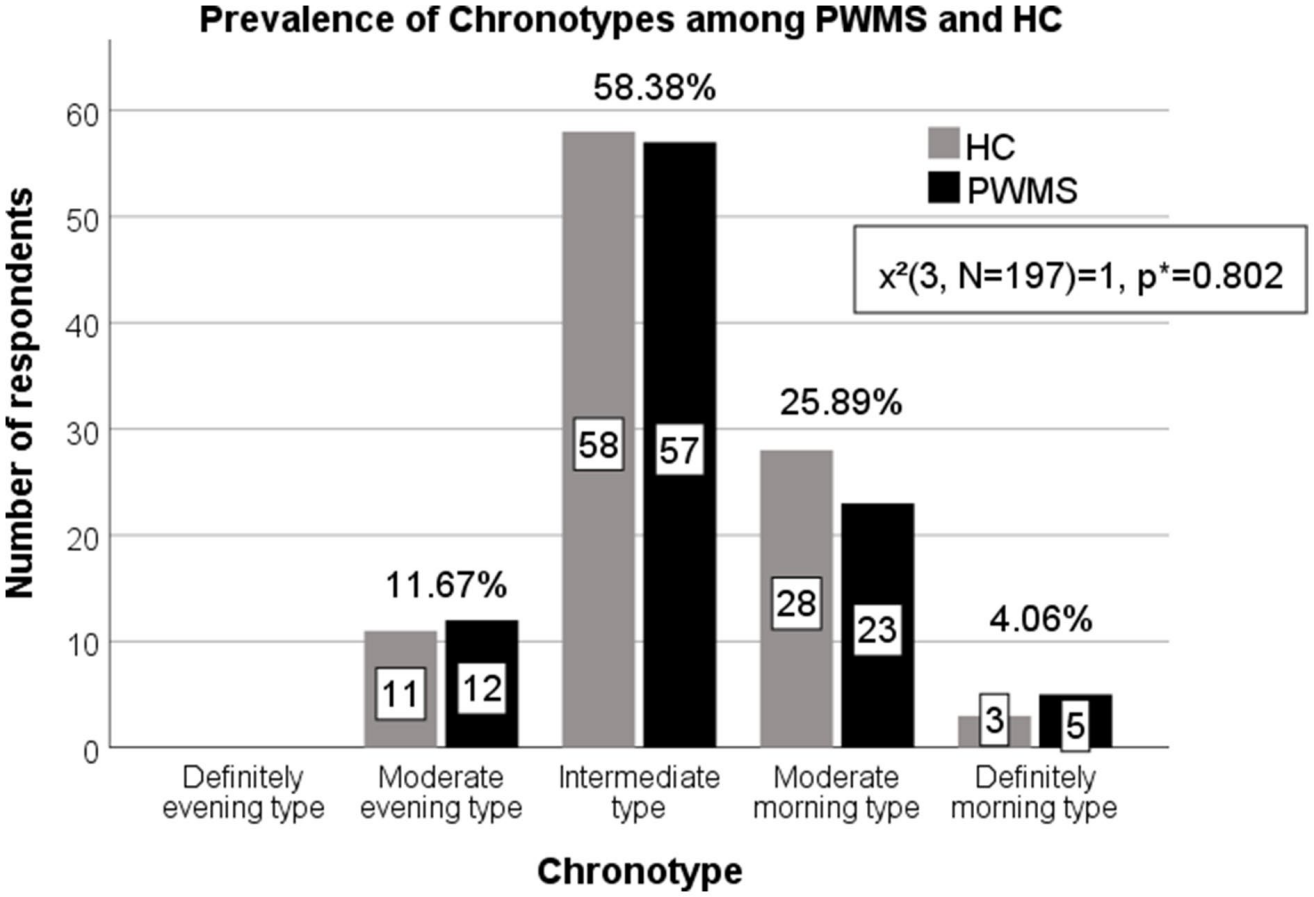
Prevalence of chronotypes among PWMS and healthy controls. PWMS, patients with multiple sclerosis; HC, healthy controls. *Chi-square test.

The average Horne-Östberg MEQ scores were 54 (IQR 15.0) and 53.5 (IQR 13.0) for PWMS and healthy controls, respectively, which were indicative of the intermediate chronotype. Most of all respondents, i.e., 115 (58.38%), belonged to the intermediate chronotype, and 51 (25.89%) – to the moderate morning type. The moderate evening and definitely morning types were less common (see Figure 3). None of the respondents belonged to the definitely evening chronotype. There was no significant difference in the distribution of chronotypes between PWMS and healthy controls (*χ*^2^(3, *N* = 197) = 1, *p* = 0.802), as well as between PWMS subgroups based on the EDSS score (*χ*^2^(3, *N* = 97) = 0.18, *p* = 0.982). However, five (62.5%) of individuals with definitely morning chronotype belonged to PWMS group.

### 3.4 Symptoms of depression, anxiety, and fatigue among different chronotype groups

Higher average HADS depression scores were observed in both PWMS and healthy controls with the moderate evening (6.25 ± 0.71) and intermediate chronotypes (5.1 ± 0.32) than in those with moderate morning chronotypes (3.86 ± 0.48) (*F*(3, 189) = 3.28, *p* = 0.022). Higher average HADS anxiety scores (9.42 ± 0.86) were observed in respondents with evening chronotypes than in respondents with other chronotypes (*F*(3, 189) = 5.59, *p* = 0.001). The average SFQ score was higher among PWMS (17.17 ± 6.24) than among healthy controls (12.84 ± 4.99) (*t*(139) = −4.34, *p* < 0.001).

**Figure 4:**
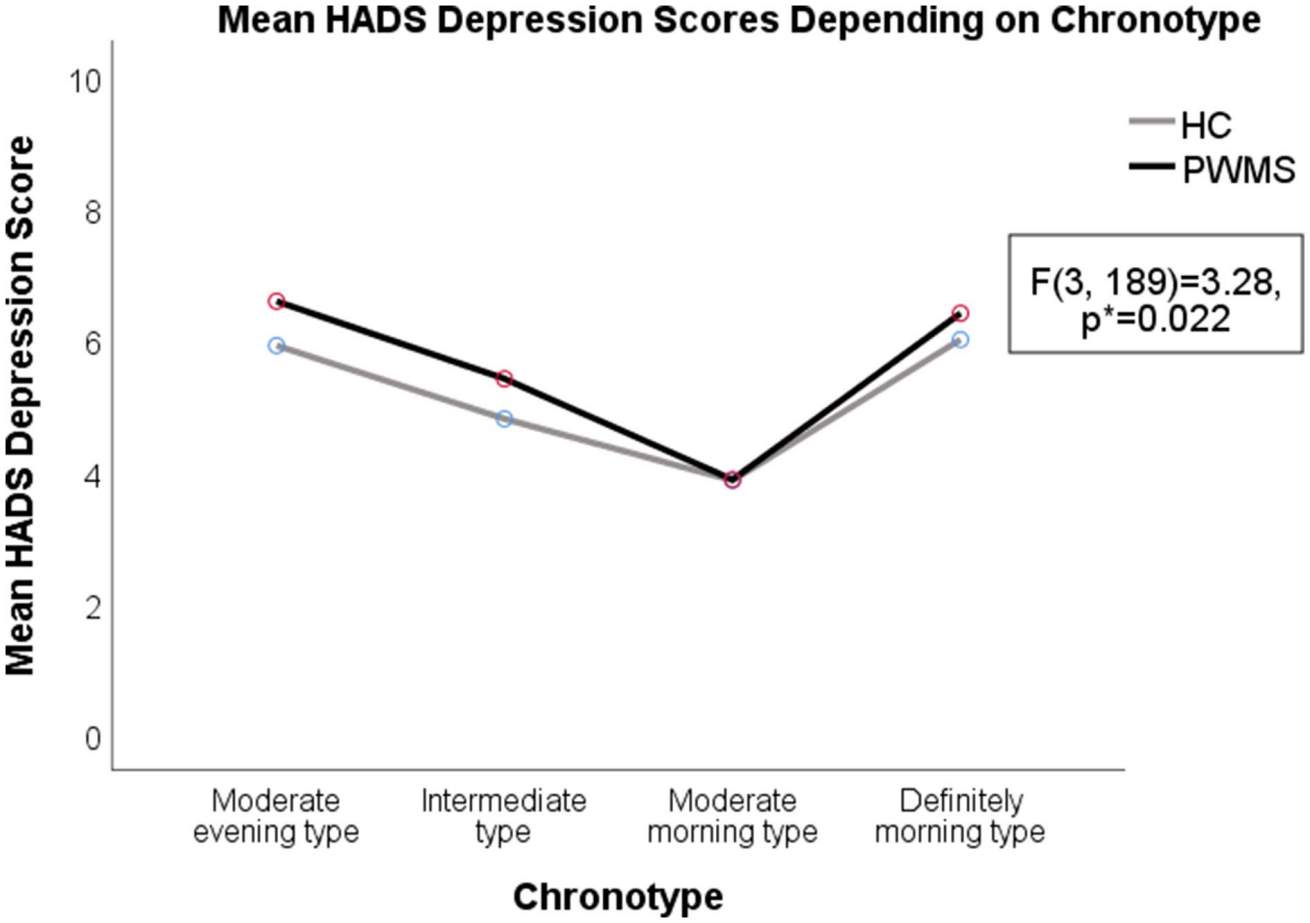
Depression symptoms in PWMS and healthy controls with different chronotypes. HADS, Hospital Anxiety and Depression Scale; HC, healthy controls; PWMS, patients with multiple sclerosis. *Two-way ANOVA.

**Figure 5:**
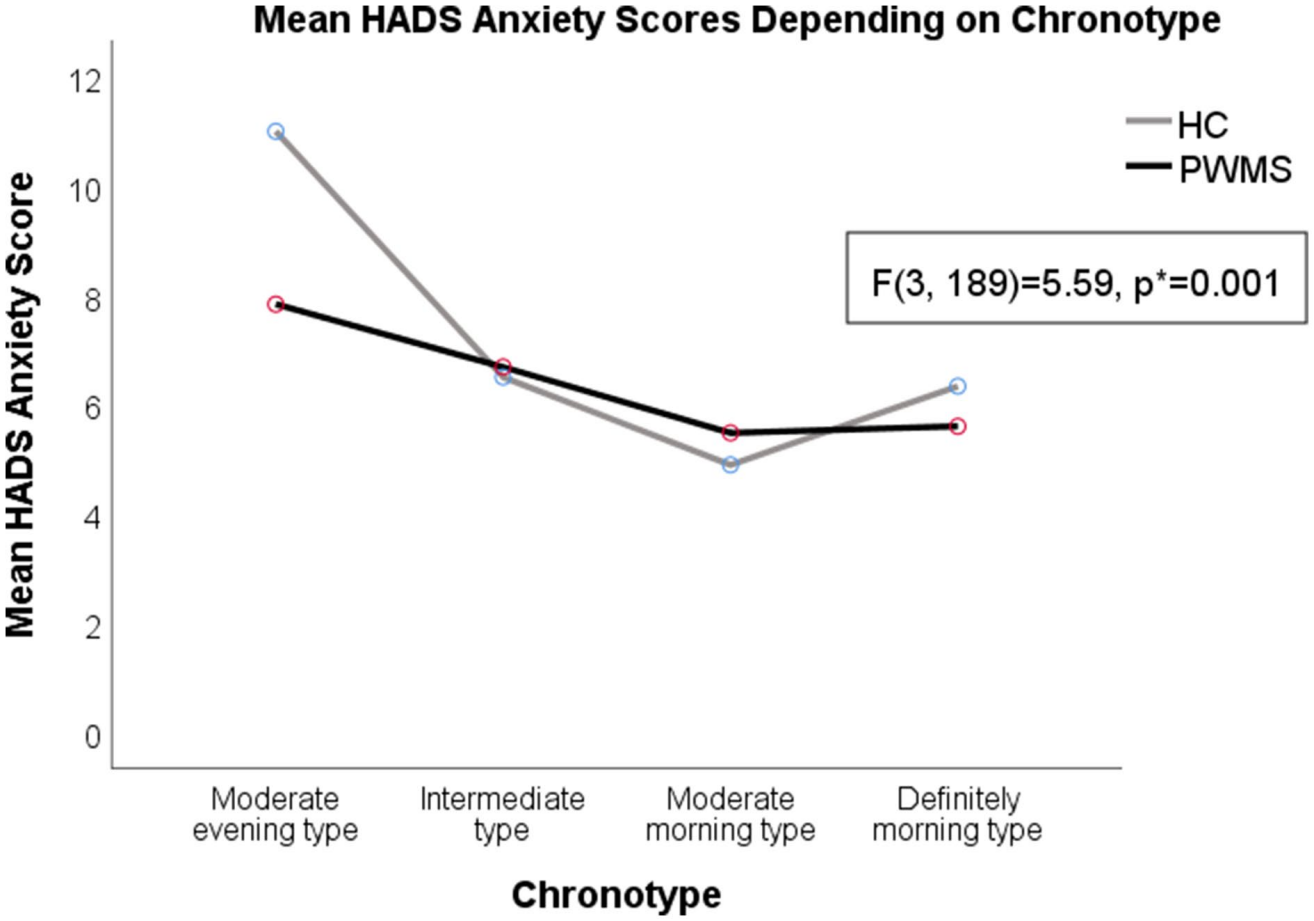
Anxiety symptoms in PWMS and healthy controls with different chronotypes. HADS, Hospital Anxiety and Depression Scale; HC, healthy controls; PWMS, patients with multiple sclerosis. *Two-way ANOVA.

**Figure 6:**
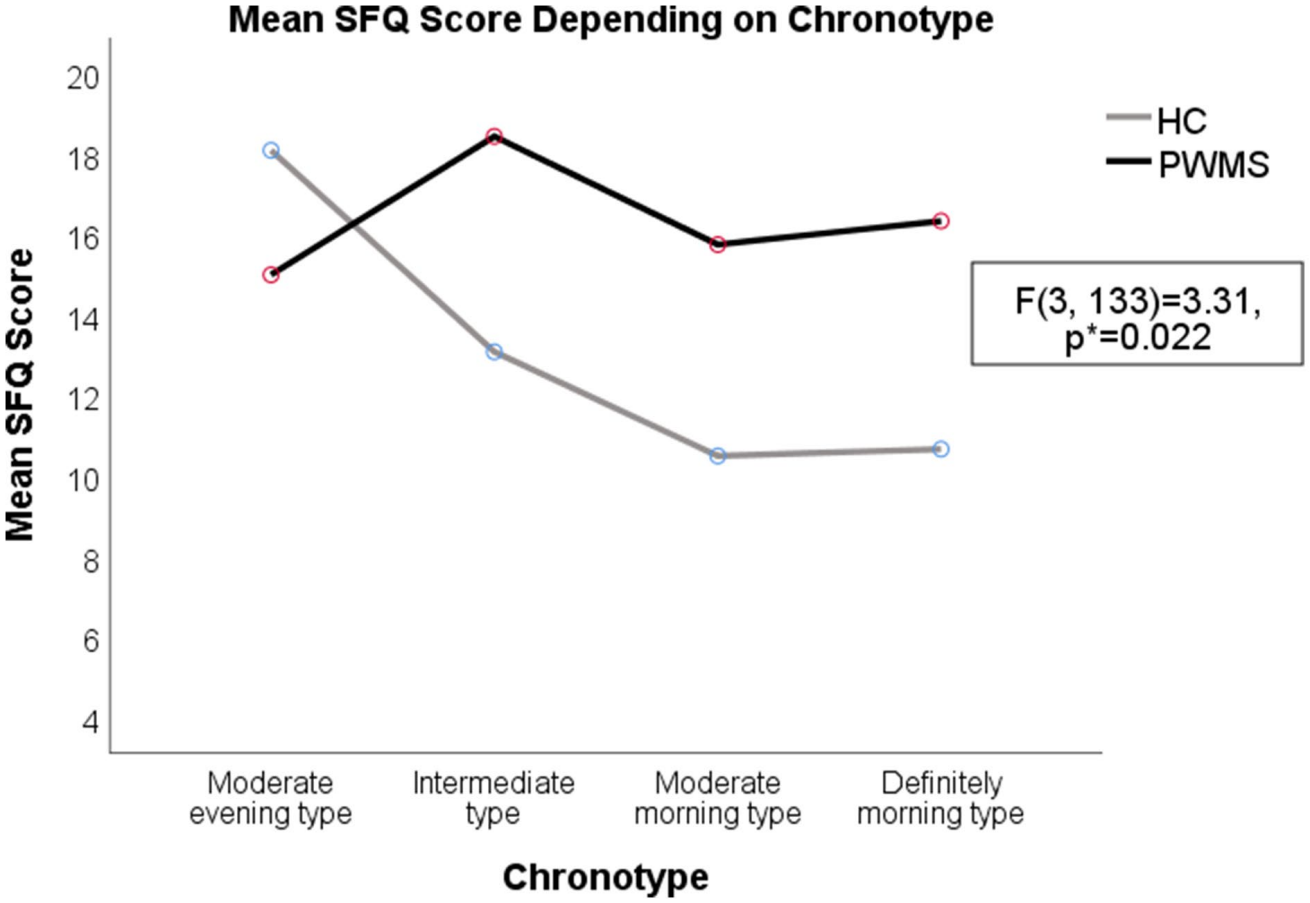
Severity of fatigue for PWMS and healthy controls with different chronotypes. HC, healthy controls; PWMS, patients with multiple sclerosis; SFQ, Shortened Fatigue Questionnaire. *Two-way ANOVA.

A two-way ANOVA revealed a significant interaction between the effects of disease and specific chronotypes on the severity of fatigue (*F*(3, 133) = 3.31, *p* = 0.022). There were higher SFQ scores among PWMS with intermediate (*F*(1, 133) = 18.1, *p* < 0.001) and morning (*F*(1, 133) = 6.59, *p* = 0.011) chronotypes, comparing to healthy controls. Fatigue levels were higher in healthy controls with evening chronotypes (*F*(3, 96) = 7.62, *p* < 0.001); however, there was no significant difference in the average SFQ score between healthy controls and PWMS with evening chronotypes (*F*(1, 133) = 1.57, *p* = 0.212; see Supplementary S2 and Figures 4–6 for details).

## 4 Discussion

Our findings revealed prolonged sleep latencies in both healthy controls and PWMS; however, PWMS took more time to fall asleep despite having increased fatigue levels. Furthermore, among PWMS, fatigue levels were positively correlated with sleep latency. A meta-analysis of quantitative sleep parameters in healthy individuals indicated that an adult should fall asleep within 15–20 min (35, 36). Our findings are consistent with previous reports of difficulties in falling asleep among PWMS, especially those with severe fatigue, and that insufficient sleep length caused more tiredness during the daytime (26, 37). Furthermore, we observed prolonged sleep latencies among PWMS who presented depression and anxiety symptoms. It was previously suggested that prolonged sleep latency and shorter sleep duration are conditioned not by solitary factors, but by expressed fatigue, concomitant affective disorders, undiagnosed sleep disorders (such as insomnia, restless legs syndrome, etc.) and MS combination (21).

In our study, PWMS tended to take naps during the day more often than healthy controls; further, the tendency to nap during the daytime was related to the severity of MS disability. Decreased night sleep duration and quality are associated with sleepiness during the day; however, daytime naps can make it difficult to fall asleep in the evening; therefore, sleep latency prolongs (26). Worsened night sleep quality among PWMS could be attributed to prolonged sleep latency as well as loss of sleep integrity (linked to spasticity-induced pain, nocturia, dysesthesia, and symptoms of restless legs syndrome); internal biological clock shifts to morning chronotypes [linked to demyelination in the SCN area (23)]; use of anti-depressives, which causes REM sleep phase disturbance; and other factors (21, 23, 38).

In our study, most PWMS belonged to the intermediate chronotype, which is consistent with the previous report by Silva et al. that the intermediate chronotype is found in approximately 60% of the adult population (15). However, other small studies have indicated that morning chronotypes are more common among PWMS than among healthy controls (29, 30, 39). In our study, only 28 (28.87%) PWMS showed the morning chronotypes (both moderate and definitely). Barun et al. reported that the morning chronotype was more common among patients with secondary progressive MS than among patients with relapsing-remitting or primary progressive MS (30). All the patients in our study had relapsing MS.

The evening chronotype for both PWMS and healthy controls, as well as the definitely morning chronotype for PWMS, were associated with both negative psychological (anxiety and depression symptoms) and physical (fatigue symptoms) effects. Our findings suggested that the moderate morning chronotype for both PWMS and HC was associated with lower depression and anxiety scores, comparing to other chronotypes. We think that moderate morning chronotype could harmonize with an individual’s social life regimen better that the evening chronotypes. On the other hand, an Italian study suggested different explanation: higher early-morning motor activities in patients with relapsing-remitting MS could be attributed to increased activity of the hypothalamus–pituitary gland-adrenal cortex axis and higher morning cortisol levels in PWMS (16, 39). Their hypothesis might partly explain the higher prevalence of MS in geographic regions that use summer time (or daylight saving time), since it may disturb the circadian rhythm balance (40).

Assessing PWMS chronotype may be useful in confirming patient’s circadian preference, furthermore, asking questions about patient’s complains of insomnia, impairment of social, occupational, or other areas of functioning could lead a neurologist to the suspicion of circadian rhythm disorder for PWMS. It was suggested that changes in the circadian rhythm may promote the pathogenesis of MS due to decreased melatonin levels and its insufficient antioxidative and anti-inflammatory effects (24, 25). Moreover, disturbances in sleep homeostasis among PWMS are related to impaired short-term memory and other cognitive functions, attention deficits, increased disability, and comorbidities; therefore, it is important to detect, diagnose, and treat sleep disorders, including circadian, in PWMS (38, 41). Circadian rhythm disorders can be treated through cognitive behavioural therapy, lifestyle changes (e.g., social life and worktime adjustments to individual chronotypes), and sleep hygiene (regular sleep regimes, dark and quiet sleep environments, etc.). In severe cases, bright light therapy and melatonin or melatonin receptor agonists are recommended (28, 42, 43).

At the end of our study, all PWMS received sleep hygiene recommendations as the first step toward improving their sleep quality. Moreover, all our patients had an opportunity to consult with sleep medicine specialists and, if necessary, to undergo more elaborate sleep studies, for example, polysomnography (44). Additionally, PWMS with symptoms of depression and anxiety were offered to be referred to a psychiatrist’s consultation in our Center.

This study had several limitations, including a moderate sample size and possible referral bias since our hospital is a tertiary referral centre. Therefore, future large-scale studies including more patients from tertiary referral centres in other Lithuanian cities are warranted. It is also important to include more patients with secondary progressive and other MS types in the future studies to search for differences in sleep habits and chronotypes according to MS type. Moreover, future studies could incorporate data from actigraphy and polysomnography, the former being important for evaluation of sleep and wake patterns in patients with circadian rhythm disorders, the latter being the gold standard for assessing sleep parameters; as well as objective methods for determining individual chronotypes, including dim-light melatonin onset.

## 5 Conclusion

This is the first Lithuanian study to evaluate the distribution of chronotypes among PWMS and healthy individuals. The intermediate chronotype was the most common chronotype among both PWMS and healthy controls. Despite significantly prolonged sleep latencies in PWMS, especially those experiencing fatigue symptoms, PWMS usually slept less at night and tended to take daytime naps. For all respondents, higher average HADS depression and anxiety scores were associated with a moderate evening chronotype. Fatigue was more commonly found for healthy controls with evening chronotype, while in PWMS group it was more prevalent for patients with intermediate and morning chronotypes. Future large-scale studies including more PWMS with secondary progressive and other MS types are needed.

## Data availability statement

The original contributions presented in the study are included in the article/Supplementary Material, further inquiries can be directed to the corresponding author.

## Ethics statement

The studies involving humans were approved by Bioethics Committee of the Vilnius University Hospital Santaros Klinikos (no. 18-429). The studies were conducted in accordance with the local legislation and institutional requirements. The participants provided their written informed consent to participate in this study.

## Author contributions

IJ: Conceptualization, Data curation, Formal analysis, Investigation, Methodology, Project administration, Software, Validation, Visualization, Writing – original draft, Writing – review & editing. ES-J: Conceptualization, Data curation, Formal analysis, Investigation, Methodology, Project administration, Resources, Supervision, Validation, Visualization, Writing – review & editing. RK: Conceptualization, Data curation, Investigation, Project administration, Resources, Supervision, Writing – review & editing. NG: Investigation, Software, Supervision, Validation, Writing – review & editing. IS: Investigation, Supervision, Validation, Writing – review & editing. JL: Investigation, Supervision, Validation, Writing – review & editing. GK: Formal analysis, Project administration, Supervision, Validation, Writing – review & editing. DJ: Formal analysis, Project administration, Supervision, Validation, Writing – review & editing.
