# Supplementary material for: Chronotypes and their relationship with depression, anxiety, and fatigue among patients with multiple sclerosis in Vilnius, Lithuania
Source: Front Neurol. 2023 Nov 27;14:1298258. doi: 10.3389/fneur.2023.1298258 (PMC10711075; doi:10.3389/fneur.2023.1298258)
Supplement: Supplementary file 1 [file Data_Sheet_1.pdf]

Supplement 1. Questionnaire about sleep habits (part of general questionnaire): original version and authors‘ translation

Chronotipų paplitimas tarp išsėtine skleroze sergančių pacientų
Prevalence of Chronotypes among Patients with Multiple Sclerosis

**Klausimynas apie miego įpročius (įeina į bendrąjį klausimyną)**	**Questionnaire about sleep habits (part of general questionnaire). Authors‘ translation**
Įrašykite, koks yra Jūsų vidutinis:<br>- atsigulimo į lovą laikas:............................<br>- užmigimo laikas: ....<br>- pabudimo laikas: ....<br>- atsikėlimo iš lovos laikas:.......................	Insert your average daily:<br>- going to bed time: ....<br>- falling asleep time: ....<br>- waking up time: ....<br>- getting out of bed time: ....
Kiek valandų **per naktį** dažniausiai miegate?<br>- <5 valandas<br>- 5 – 7 valandas<br>- 8 – 10 valandų<br>- >10 valandų	How many hours **per night** do you usually sleep?<br>- <5 hours<br>- 5 – 7 hours<br>- 8 – 10 hours<br>- >10 hours
Ar miegate dienos metu?<br>- taip<br>- ne	Do you take naps during the day?<br>- yes<br>- no
Jei taip, kiek valandų miegate **dienos metu**?<br>- <30 min.<br>- 30 min. – 1 val.<br>- 1 – 2 val.<br>- >2 val.	If yes, how many hours do you sleep **during the day**?<br>- <30 minutes<br>- 30 minutes – 1 hour<br>- 1 – 2 hours<br>- >2 hours
Kiek valandų dažniausiai miegate **per parą**?<br>- <5 valandas<br>- 5 – 7 valandas<br>- 8 – 10 valandų<br>- >10 valandų	How much time do you usually sleep **per 24-hours**?<br>- <5 hours<br>- 5 – 7 hours<br>- 8 – 10 hours<br>- >10 hours
Ar savo miego aplinką (čiužinio minkštumą, kambario tamsumą, aplinkos ramumą ir kt.) įvertintumėte kaip patogią miegui?<br>- taip<br>- ne	Would you rate your sleeping environment (softness of the mattress, darkness of the room, quietness of the environment, etc.) as comfortable for sleep?<br>- yes<br>- no
Kokie **išoriniai** aplinkos veiksniai (pvz., triukšmas, karštis, šaltis, intensyvi šviesa, garsai ir pan.) Jums trukdo užmigti ir palaikyti stabilų miegą naktį (įrašykite)?<br>..<br>..	What **external** environmental factors (such as noise, heat, cold, intense light, sounds, etc.) prevent you from falling asleep and maintaining stable sleep at night (write down)?<br>..<br>..
Kokie **vidiniai** veiksniai (t.y., nuo Jūsų priklausomi, pvz., nerimas, įkyrios mintys, jaudinimasis dėl kitos dienos, nuovargio nebuvimas prieš miegą ir pan.) Jums trukdo užmigti ir palaikyti stabilų miegą naktį (įrašykite)?<br>..<br>..	What **internal** factors (i.e., that dependent on you, such as anxiety, intrusive thoughts, worrying about the next day, not being tired before bed, etc.) prevent you from falling asleep and maintaining stable sleep at night (write down)?<br>..<br>..

Ar šiuo metu vartojate nuotaiką koreguojančius vaistus (antidepresantus)?<br>- taip<br>- ne	Are you currently taking mood-altering medications (antidepressants)?<br>- yes<br>- no
Ar vartojate priemones miego sutrikimų gydymui?<br>- taip<br>- ne	Do you take remedies for sleep disorders?<br>- yes<br>- no
Ar vartojate migdomuosius preparatus (hipnotikus)?<br>- taip<br>- ne<br>Jei atsakėte „taip“, įrašykite konkrečius vaistus:<br>..	Do you use sleeping pills (hypnotics)?<br>- yes<br>- no<br>If yes, please list the specific medications:<br>..
Ar šiuo metu vartojate nerimą mažinančius vaistus (benzodiazepinus)?<br>- taip<br>- ne<br>Jei taip, kokius?...	Are you currently taking anti-anxiety medication(s) (such as benzodiazepines)?<br>- yes<br>- no<br>If yes, please list the specific medications:<br>..
Ar budrumo didinimui dienos metu vartojate:<br>- kofeiną<br>- energetinius gėrimus<br>- išvardintų priemonių nevartoju<br>- vartoju kitus preparatus (įrašykite)<br>...	Do you use some of these substances during the day to increase your alertness?<br>- caffeine<br>- energy drinks<br>- I do not use any of these<br>- I use other substances (write down):<br>...
Kiek puodelių kavos vidutiniškai išgeriate per dieną? (įrašykite puodelių skaičių)<br>...	How many cups of coffee do you drink per day (on average; enter the number of cups)?<br>...
Ar miego gerinimui vartojate alkoholį (vakare/prieš pat miegą)?<br>- taip<br>- ne	Do you use alcohol to improve sleep (in the evening/before bed)?<br>- yes<br>- no
Jei taip, kaip dažnai vartojate alkoholinius gėrimus prieš eidamas/a miegoti?<br>- kas vakarą<br>- 4 – 6 kartus per savaitę<br>- 2 – 3 kartus per savaitę<br>- 1 kartą per savaitę<br>- rečiau nei 1 kartą per savaitę	If yes, how often do you drink alcoholic beverages before going to bed?<br>- every evening<br>- 4 – 6 times per week<br>- 2 – 3 times per week<br>- 1 time per week<br>- < 1 time per week

Dėkojame už atsakymus!

Thanks for your answers!
