# Supplementary material for: Chronotypes and their relationship with depression, anxiety, and fatigue among patients with multiple sclerosis in Vilnius, Lithuania
Source: Front Neurol. 2023 Nov 27;14:1298258. doi: 10.3389/fneur.2023.1298258 (PMC10711075; doi:10.3389/fneur.2023.1298258)
Supplement: Supplementary file 2 [file Data_Sheet_2.pdf]

Supplement 2. Symptoms of Depression, Anxiety, and Severity of Fatigue in PWMS and Healthy Controls with Different Chronotypes

Chronotype	Subgroups	F value	Mean	SD	Partial Eta Squared	**p-value
HADS Depression Score***						
Moderate evening type	Both*	3.28	6.25	±0.71	0.05	0.006
Moderate morning type			3.86	±0.48		
Intermediate type	Both*	3.28	5.1	±0.32	0.05	0.032
Moderate morning type			3.86	±0.48		
HADS Anxiety Score***						
Moderate evening type	Both*	5.59	9.42	±0.86	0.81	0.003
Intermediate type			6.59	±0.39		
Moderate evening type	Both*	5.59	9.42	±0.86	0.81	<0.001
Moderate morning type			5.19	±0.58		
Moderate evening type	Both*	5.59	9.42	±0.86	0.81	0.048
Definitely morning type			5.97	±1.51		
Intermediate type	Both*	5.59	6.59	±0.39	0.81	0.045
Moderate morning type			5.19	±0.58		
SFQ Score***						
Intermediate type	PWMS	18.1	18.44	±1.06	0.12	<0.001
	HC		13.09	±0.67		
Moderate morning type	PWMS	6.59	15.75	±1.81	0.05	0.011
	HC		10.5	±0.96		

HADS, Hospital Anxiety and Depression Scale; HC, healthy controls; PWMS, patients with multiple sclerosis; SFQ, Shortened Fatigue Questionnaire; SD, standard deviation.

* There was no significant difference between PWMS and HC within different chronotype groups; therefore, we report only the differences between the chronotype groups.

**Two–way ANOVA and *post hoc* Least Significant Difference (LSD).

***All the other comparisons between chronotype groups were not statistically significant.
